# Optimization and prediction of tribological behaviour of filled polytetrafluoroethylene composites using Taguchi Deng and hybrid support vector regression models

**DOI:** 10.1038/s41598-022-14629-5

**Published:** 2022-06-21

**Authors:** Musa Alhaji Ibrahim, Hüseyin Çamur, Mahmut A. Savaş, S. I. Abba

**Affiliations:** 1 Mechanical of Engineering Department, Faculty of Engineering, Kano University of Science and Technology, Wudil KM 50, Kano, Gaya Road, Wudil P.M.B 3244, Kano, Kano, Nigeria; 2 Mechanical Engineering Department, Faculty of Engineering, Near East University, via Mersin 10, 99138, Nicosia, Turkey; 3 Interdisciplinary Research Center for Membrane and Water Security, King Fahd University of Petroleum and Minerals, Dhahran, 31261 Saudi Arabia

**Keywords:** Mechanical engineering, Theory and computation

## Abstract

This study presents optimization and prediction of tribological behaviour of filled polytetrafluoroethylene (PTFE) composites using hybrid Taguchi and support vector regression (SVR) models. To achieve the optimization, Taguchi Deng was employed considering multiple responses and process parameters relevant to the tribological behaviour. Coefficient of friction (µ) and specific wear rate (K_s_) were measured using pin-on-disc tribometer. In this study, load, grit size, distance and speed were the process parameters. An L_27_ orthogonal array was applied for the Taguchi experimental design. A set of optimal parameters were obtained using the Deng approach for multiple responses of µ and K_S_. Analysis of variance was performed to study the effect of individual parameters on the multiple responses_._ To predict µ and Ks, SVR was coupled with novel Harris Hawks’ optimization (HHO) and swarm particle optimization (PSO) forming SVR-HHO and SVR-PSO models respectively, were employed. Four model evaluation metrics were used to appraise the prediction accuracy of the models. Validation results revealed enhancement under optimal test conditions. Hybrid SVR models indicated superior prediction accuracy to single SVR model. Furthermore, SVR-HHO outperformed SVR-PSO model. It was found that Taguchi Deng, SVR-PSO and SVR-HHO models led to optimization and prediction with low cost and superior accuracy.

## Introduction

Filled polymer matrix composites (PMCs) containing fillers continue to receive significant attention from academics and industries due to their modified mechanical and tribological behaviours than virgin polymers^1^. Polymer based composites showed improved tribological resistance^2^. Of the different kinds of polymers, polytetrafluoroethylene (PTFE) filled with carbon or bronze fibres are widely used due to their high mechanical and low tribological behaviour^3^. It has been indicated that these composites are suitable in sectors where mechanical parts including brakes and clutches tribological behaviours are significant^4–7^. It has been generally agreed that tribological resistance of materials can be improved by adding more filler content to a certain limit^8,9^ to neat polymers. Polytetrafluoroethylene (PTFE) has been one of the commonly used thermoplastic matrices for wear conditions because of its low coefficient of friction, ease of process-ability, chemical inertness, low density and low-cost^10,11^.

Wear is one of the most commonly encountered problems in industries causing frequent substitution of parts especially abrasion. Abrasive wear of various polymers and filled polymers have been studied experimentally. Abrasive wear rate of different matrices were studied by^12^ and it was found that different polymer exhibited dissimilar wear rate. Inclusion of glass and carbon fabric into vinyl/ester were analyzed. It was reinforced vinyl/ester combination indicated lower wear rate than glass and/or carbon fabric reinforced vinyl/ester composite^13^. As reported by^14^ applied load found as the most significant process parameter; reduced wear rate was observed when performance UHMWPE was reinforced with fillers. According to^15^ it seen that mass loss and µ increased with increase in speed and decrease in grit sizes for betelnut filled epoxy composites.

In order to study multiple responses related to tribological behaviours of composites several decision-making methods including data development, analytic hierarchy as well as grey relational analysis (GRA) have been proposed in the literature^16^. Of these models, GRA proposed by Deng in 1989 is the widely used methodology especially when the nature of the information is not certain and complete^17^. Dharmalingam, Subramanian and Kok combined grey relational analysis (GRA) with Taguchi to optimize abrasive tribological property of aluminium hybrid metal composites. Analysis of variance (ANOVA) indicated that grit size was the parameter that had the most influence on wear rate and load was found to had the greatest effect on coefficient of friction^18^. Sylajakumar et al.^19^ used Taguchi-GRA method to study the effect of load, speed and distance on coefficient of friction and wear rate of co-long composite. ANOVA showed that speed significantly affect the wear property of the co-continuous composite. Savaran and Thanigaivelan^20^ optimized dimple geometry and laser parameter using principal component analysis (PCA) coupled GRA. ANOVA showed that average power contributed most while depth contributed less to performance measures. An integrated Taguchi OA and GRA method has been applied to optimize injection moulding parameters of HDPE-TiO2 nanocomposites Pervez et al.^21^. The work established that optimum parameters were content of TiO_2_ at 5%, barrel temperature of 225 °C, residence time of 30 min and holding time of 20 s. Adediran et al. optimized mechanical properties of hybrid propylene reinforced bio composites using Taguchi model. It was found that collage of 4% PSS and 10% kenaf fibre produced the optimum combination for hybrid bio composites^22^. Besides this, Taguchi method hybridized with grey relational grade has as well been employed for multi-response optimization of wire discharge electrical discharge machining^23^, turning process^24^ and milling parameter^25^.

Due to nonlinearity and complex nature of tribological behaviour of materials soft computing methods are increasingly widely accepted including support vector machine (SVM), adaptive neuro-fuzzy inference system (ANFIS) and artificial neural network (ANN). The reason being the fact that these models are capable of capturing the nonlinear and complex nature of the relation between the tribological parameters and responses as compared to conventional mathematical techniques at much cheaper running costs. Various forms of wear are encountered such as abrasion, adhesive, fretting and fatigue wear. Abrasive tribology for composites, instruments, coatings, hip implant, airplane manufacturing as well as automotive components are of essential importance as it determines parts’ performance or longevity. This in general is checked experimentally, as process parameters such as materials characteristics, surface texture, sliding speed and sliding speed. In the analysis of tribology, many mathematical modeling methods have been built. Among them are atomic and molecular kinetics, finite element method, symptom modelling, continuum mechanics, dimension reduction, analysis, boundary element system, stochastic models^26^. Nevertheless, since tribological behaviours are complex and nonlinear, mathematical models are limited.

Lately, the use artificial intelligent (AI) models has become widely accepted in tribology. Jones et al. pioneered the use of ANN to predict life data and tribological behaviours. Accurate prediction of tribological property by ANN gives an option to the present time, cost and energy consuming testing approaches. Since then, the method has been successfully applied in the tribology discipline that includes wear of reinforced polymer composites^27,28^, coefficient of friction and mechanical properties, respectively^29,30^, compensation of magnetic levitation using ANN based on fuzzy inference^31^. ANFIS and ANN were compared in the prediction of K_s_ PTFE and its composites. It was found that ANN performed better than ANFIS^32^. Prediction of abrasive wear of industrial waste and glass filled polyester composites was done using ANN and linear regression model. The results found that ANN outperformed the linear model^33^. SVM has been employed in the prediction of tool wear appraisal^34,35^. Also SVM, RBFF and ANN have been contrasted to predict diameter of PCL/gelatin materials. It was reported that ANN did better than SVR and RBFF put together^36^. Response surface methodology, ANN-HHO as well as model was used in the prediction of abrasive wear of ultrahigh strength martensitic steel. It was reported that hybridized ANN-HHO showed better performance than the single ANN model^37^.

**Figure 1:**
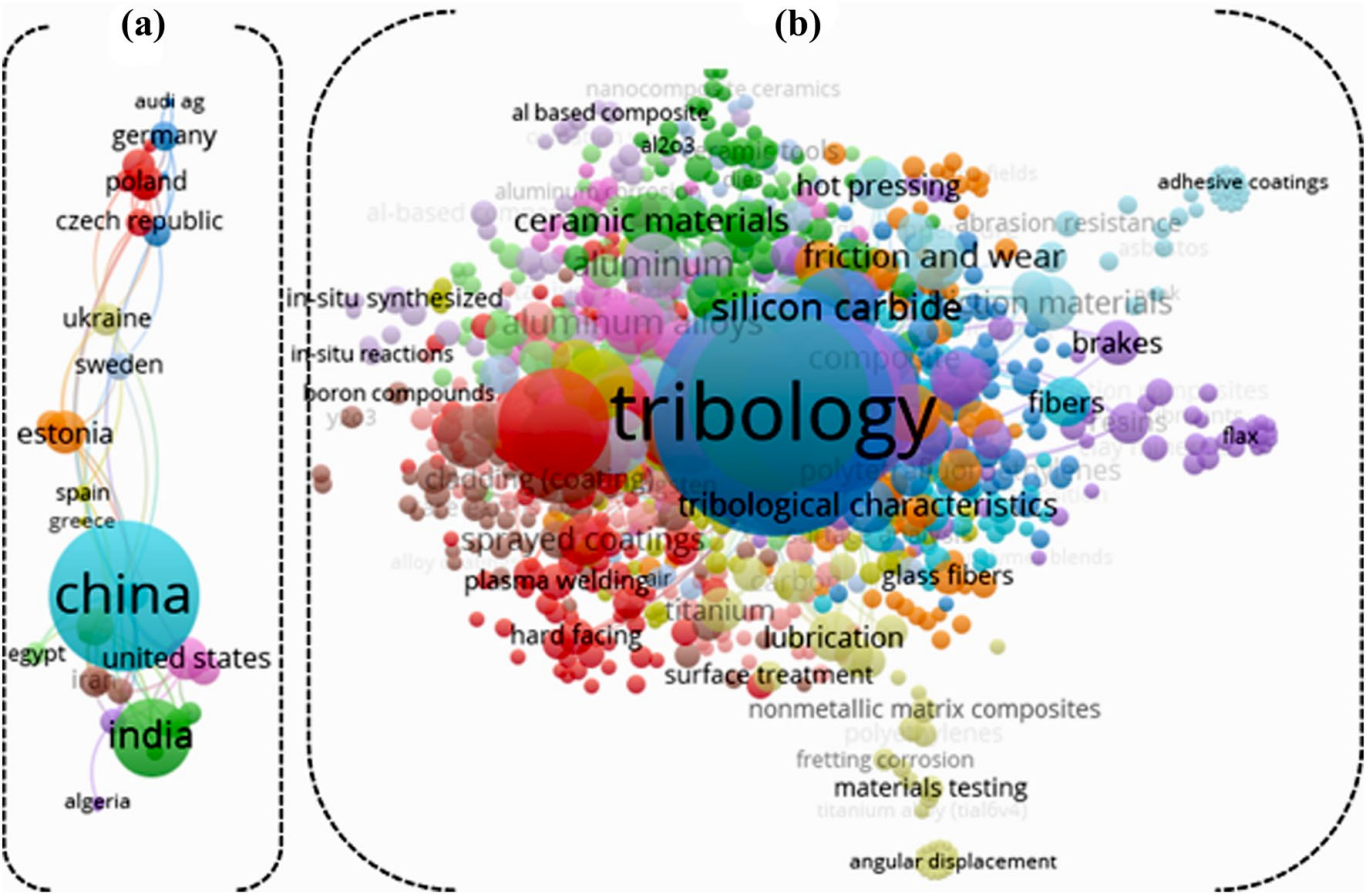
(**a**) Major keywords used over the literature on the abrasive tribology of PTFE based composites field (1989–2021), (**b**) the investigated the research region for abrasive.

A survey of the reported literature on the database Scopus yielded the findings that there were 450 peer-reviewed papers starting from 1989 up to date adopted over the literature using the feasibility of wide interest for the abrasive tribological behaviour of PTFE based composites. Figure 1b shows 388 keywords occurrence between those studies, indicating the deep interest and implementation of this field. In addition, the popularity of this study topic was investigated in different regions throughout the world, with the bulk of the countries producing the output being China, the United States, and India. (Fig. 1a). The motivation of this study demonstrated excellent AI techniques for predictions abrasive tribological behaviour of filled PTFE composites. Generally each study has progressed to a little higher degree of accuracy for observations and efficiency at a deeper level than the previous one. To the best knowledge of the authors, no study published in a technical literature has predicted the abrasive tribological behaviour of filled PTFE composites employing this approach using small amount of data. As a result, the goal of this work is to optimize and predict multi-response variable of coefficient of friction (µ) and specific wear rate (K_s_) of abrasive wear of reinforced PTFE composites using Taguchi Deng and novel hybrid support vector regression (SVR) model.

## Results and discussion

### Experimental results

The results of the tribological experiments for the various test conditions are shown in Table 1. It was seen that the tribological behaviours of the samples indicated rise and fall trend with varying the parameters. Each trial was performed twice and the average was used for the calculations. Highest SNRs of µ and K_s_ occurred at 20 and 12 trials, respectively. These give the minimum tribological rate of the filled PTFE composites. In spite of the fact the temperature was not computed, the temperature increased as the sliding distance increased. Data in Table 1 was used for the calibration and validation of the SVR, SVR-PSO and SVR-HHO models.

**Table 1:** Experimental results and their corresponding SNRs of filled PTFE composites based on Taguchi ${L}_{27}$(3^4^) OA.

Trial	Experimental results	Experimental results	Signal to noise ratio	Signal to noise ratio
	µ	Ks (mm^3^N^−1^ m^−1^)	µ SNR(dB)	Ks (dB)
1	0.1115	8.5700E−06	19.05	101.74
2	0.2485	1.3333E−05	12.09	97.91
3	0.1695	9.8361E−06	8.83	92.07
4	0.1930	1.7284E−05	14.29	95.65
5	0.2790	2.2584E−05	11.09	93.34
6	0.2865	6.9217E−06	28.18	103.82
7	0.1335	1.1977E−05	17.49	98.84
8	0.2265	1.7443E−05	12.90	95.58
9	0.5330	1.8281E−05	10.57	91.08
10	0.2735	4.6737E−06	11.26	107.01
11	0.2025	1.5357E−06	13.87	116.69
12	0.2220	3.8251E−06	10.46	115.86
13	0.3035	3.4632E−06	10.36	109.61
14	0.1265	6.7258E−06	17.96	103.86
15	0.9595	6.2096E−06	13.07	112.50
16	0.1115	2.1261E−05	19.05	93.85
17	0.5565	2.8293E−05	5.09	91.38
18	0.4475	1.9563E−05	22.38	88.89
19	0.3680	2.9341E−06	8.68	111.05
20	0.0335	2.9071E−06	29.50	111.14
21	0.0490	2.0864E−06	12.18	116.02
22	0.0855	9.5238E−06	21.36	100.83
23	0.1155	1.2683E−05	18.75	98.35
24	0.2805	8.3789E−06	12.08	104.74
25	0.4025	6.0553E−06	7.90	104.76
26	0.2885	1.1623E−05	10.80	99.11
27	0.3075	1.0403E−05	14.33	110.76

#### Effect of load on K_s_ and µ

**Figure 2:**
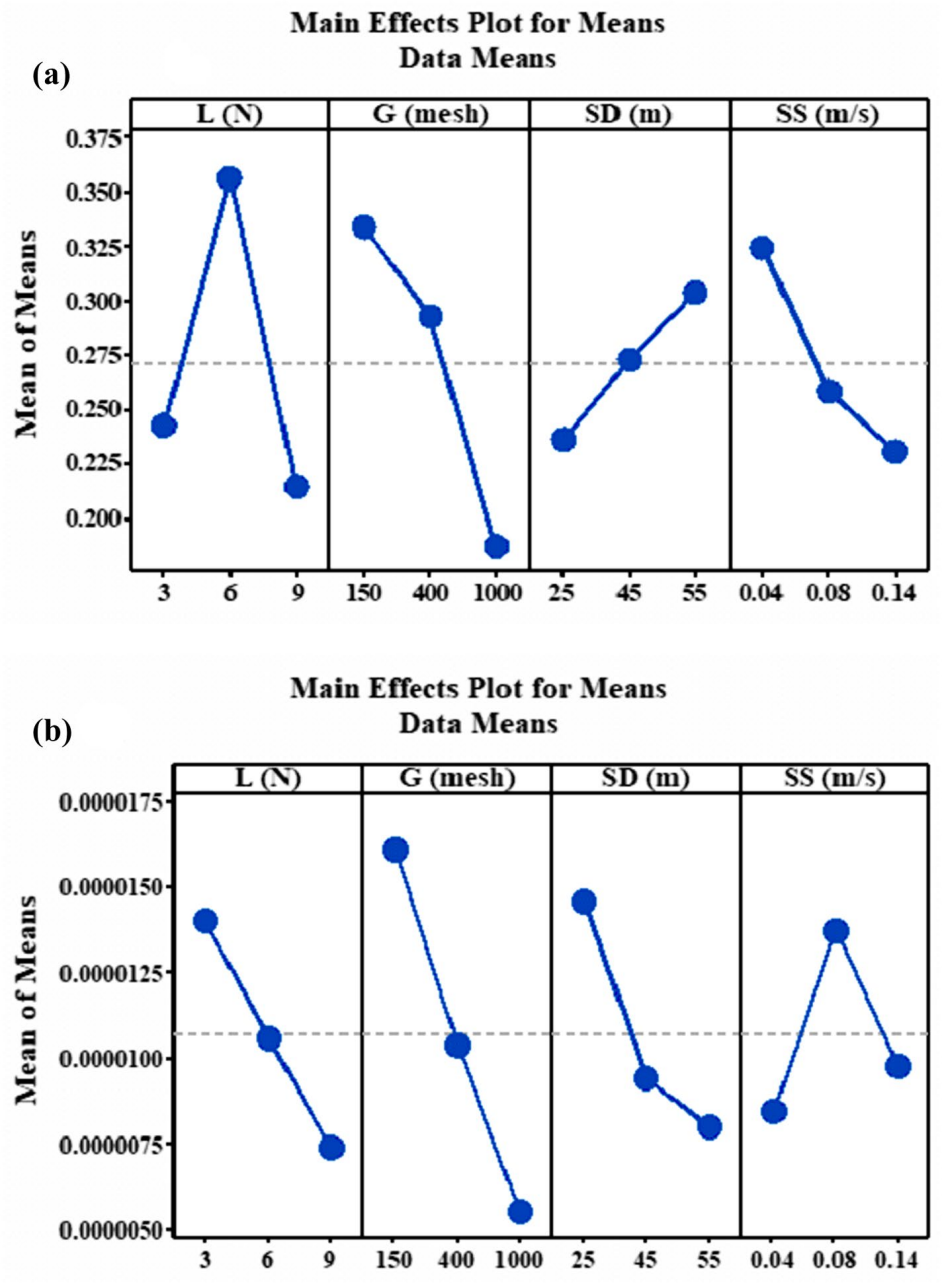
Main effect plot for mean (**a**) µ and (**b**) K_s_ of filled PTFE composites.

The results of the µ and Ks are shown in Fig. 2a,b, respectively. It was observed that as the load increase the µ and Ks decrease. The low µ at maximum load is because of the formation tribolayer by the fibres at interacting state as well as temperature and visco-elastic related behaviour. This layer prevented the pin samples to be in direct contact with the abrasive surface. Similar observation found in^38^ when wear rate and coefficient of friction of plastic reinforced glass fibre was studied against different rough and mild steel surfaces. In the work, the results showed that µand Ks decreased as the load increased from 10 to 15 N. At 6 N µ was high perhaps due to tearing of the fibre tribo layer at the contact region. This finding was contrary to results obtained by^39^ when abrasive wear of reinforced carbonized bone ash particulate polypropylene was investigated. It was found that Ks increased as the load increased from 5 to 15 N. K_s_ was low at high load because of great increase in apparent contact area at higher loads thereby leading to increase in contact area permitting a large number of particles to meet the interface and share the stress. This, in turn, reduced the wear rate.

### Effect of abrasive size on µ and K_s_

As shown in Fig. 2b increase in abrasive sizes decrease the both µ and K_s_. High µ at small grit size is related to high roughness of SiC particles that offered significant amount of resistance while the low µ is attributed to smoothness of the SiC particles that offered little resistance to the materials all due to formation of protective layer at contact surface. Decrease in wear rate due to increase in abrasive size is related to clogging of the wear track with wear debris and reduction in cutting efficiency of the abrasives due to transfer. Similar results were reported in^40^ where the abrasive wear performance of elastomers was investigated. In the study, different abrasive size of 82, 125, 269 and 425 µm was used as the counterface at constant speed of 0.01 m, applied load of 10 N but varying speed of 0.25, 0.5 and 1 m/min. It was observed that as the abrasive size of the SiC increased both the µ as well as Ks reduced.

### Effect of sliding distance on µ and K_s_

Figure 2a,b shows the relationship between the parameters and the µ and Ks, respectively. As observed in the figure, increasing the sliding distance increase the µ while a decrease in Ks observed. This is explained on the basis that distance acted as a lubricant to rubbing surfaces and therefore separated pin specimens from the counter front. Ks reduction due to increase in sliding distance is attributed to pull out or fracture of abrasives as a result of presence of tough fibres. Additionally, wear debris is transferred to counterface from the PTFE causing reduced wear rate. This result is validated by^41^ in which two-body abrasive wear property of silicon carbide filled glass fabric epoxy composites glass was studied. Grit sizes of 600 and 1000 mesh and sliding distance of 25, 50, 75 and 100 m were used as experimental conditions. The results revealed that Ks significantly reduced as abrading distances and grit size increased. Drastic reduction of Ks was observed at 25–50 m.

### Effect of sliding speed on µ and K_s_

As the sliding speed increase µ as well as K_s_ decrease and high µ and Ks are noticed at low speed due to samples’ increased contact time with the counterface (Fig. 2a,b. As the rotation rate increases and the samples are oxidized, the temperature at the contacting surfaces changes. This aids formation of a mechanically mixed and rough coating which is laid on the parts. This coating is impervious to removal and depreciates the µ and Ks significantly. When the surface of the counterface is less touched and the protection of the hard layer is heavily mixed tribological behaviours are reduced. It has been shown that reduction in wear rate depends on production of adherent, uniform and thin hard layer on the counter front when polyphenylene reinforced with CuO, SiC, TiO_2_ and ZnO nano particles to study the influence of these particles on the wear rate of the composites. It was found that with the addition of these particles there was a formation of adherent and uniform tribolayer between the samples and the counterface especially with 2 wt% of CuO and TiO_2_^42^. More so, it was observed in^43^ when the function of filler deformation, filler-bonding and counterface of Ag_2_S, CuS, ZnF_2_ and SnS inorganic fillers were introduced into polyphenylene sulfide. The results indicated that minimum wear achieved by Ag_2_S and CuO was due to the formation of thin, adherent and uniform transfer film formed on the counterface preventing the samples from coming into direct contact with surface while ZnF_2_ and SnS high wear rate was attributed to thick, non-uniform tribolayer between contact surface and samples. Similar observation was made in^44^ when SiC and graphite particles as secondary filler was added to epoxy polymer to study the influence of speed, distance and load on the epoxy composites. It was found that as the speed increased the wear properties of the reinforced epoxy composites decreased results. Similar result was reported by^44^ when 3 body abrasive tribological property of glass fibre reinforced polyester composites was investigated. It was found that as the speed increased the wear rate of the composites decreased at constant applied load as well as particles of 200 and 300 6 µm.

In all the analysis, it was found that addition of bronze and carbon fillers into PTFE improved the virgin PTFE’s tribological rate. This might be attributed to stiffness and hardness of the fillers. However, BF40 composites showed a slightly lower wear resistance than CF25 composites. This is explained on this basis of the higher weight percentage of the bronze particles that induced more hardness and larger size about 6 µm.

### Results using Taguchi approach

**Figure 3:**
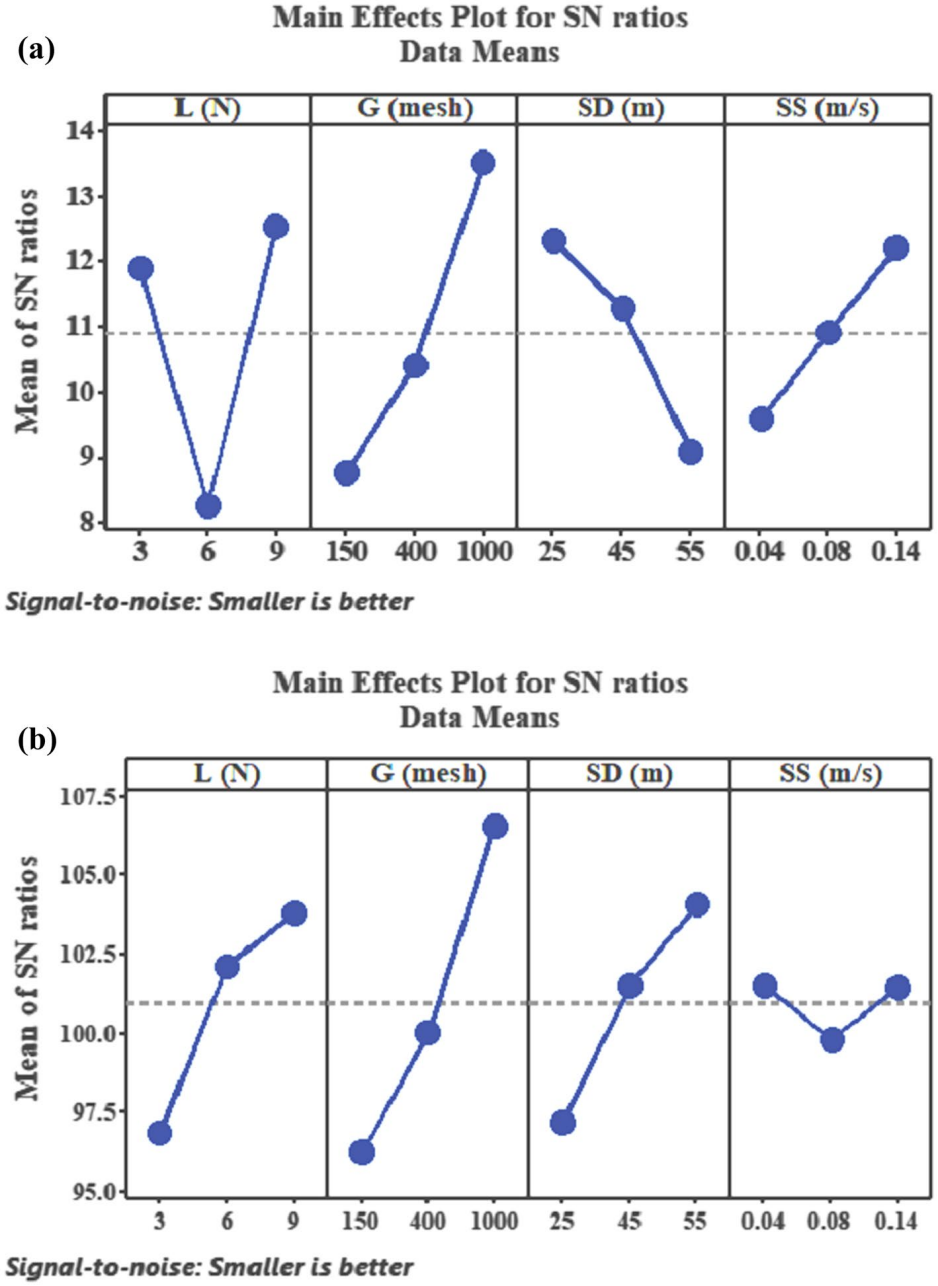
Main effect plot for SNRs of (**a**) µ and (**b**) Ks.

The experimental data in Table 1 was transformed into signal to noise ratios (SNRs) using Eq. (4) and the corresponding SNRs was as shown in Table 1. Larger SNRs indicate the minimum variation difference between wanted response and measured response. The maximum value of SNRs at the main effect plot for SNRs give the desired results. Figure 3a,b shows the mean SNRs of µ and Ks, respectively. Table 2a,b presents the computed mean SNRs for the µ and Ks, respectively. As seen in Fig. 3a, the maximum mean SNR achieved for µ were load at 9 N, grit size at 1000 mesh, sliding distance at 25 m and sliding speed at 0.14 ms^−1^. Thus, the estimated optimum parameters for achieving a minimum µ via Taguchi optimization can be coded as L3G1SD1SS3. For the Ks (Table 2b; Fig. 3b), the highest mean SNR obtained for Ks were load at 9 N, grit size at 1000 mesh, sliding distance at 55 m and sliding speed at 0.04 ms^−1^. Therefore, by Taguchi method the predicted optimum parameters are styled as L3G1SD3SS1. ANOVA depicts the parametric setting that significantly influence the abrasive behaviours. Similarly, the important parametric factor that significantly affect the µ were found as grit size followed by load, distance and speed 3(a). The percentage contribution of grit size, load, distance and speed were computed as 37.24%, 33.92%, 17.62% and 11.20%, (Table 3a). Table 2b shows the percentage contribution of the parameters on Ks. As seen grit size contributed 51.06%, load contributed 24.65% distance contributed 22.57% and speed contributed 1.72% implying that grit size most significantly influence the Ks followed by load, distance and speed, respectively.

**Table 2:** Response table for SNRs of (a) µ and (b) K_s_.

(a)					(b)			
Level	L (N)	G (mesh)	SD (m)	SS (ms^−1^)	L (N)	G (mesh)	SD (m)	SS (ms^−1^)
1	11.92	8.78	12.35	9.59	96.88	96.26	97.23	101.54
2	8.28	10.42	11.29	10.92	102.16	100.03	101.54	99.85
3	12.53	13.53	9.09	12.3	103.82	106.57	104.09	101.47
Delta	4.27	4.75	3.25	2.64	6.94	10.31	6.86	1.69
Rank	2	1	3	4	2	1	3	4

**Table 3:** Reference and deviation sequences post data processing.

Run	Reference sequence	Reference sequence	Deviation sequence	Deviation sequence
	µ	Ks	µ	Ks
1	0.9158	0.6657	0.0842	0.2629
2	0.7678	0.4395	0.2322	0.4409
3	0.8531	0.6056	0.1469	0.3102
4	0.8278	0.2518	0.1722	0.5886
5	0.7349	0.0000	0.2651	0.7866
6	0.7268	0.7441	0.2732	0.2013
7	0.8920	0.5039	0.1080	0.3902
8	0.7916	0.2442	0.2084	0.5945
9	0.4606	0.2044	0.5394	0.6258
10	0.7408	0.8509	0.2592	0.1173
11	0.8175	1.0000	0.1825	0.0000
12	0.7964	0.8912	0.2036	0.0856
13	0.7084	0.9084	0.2916	0.0720
14	0.8996	0.7534	0.1004	0.1940
15	0.0000	0.7779	1.0000	0.1747
16	0.9158	0.0624	0.0842	0.7375
17	0.7910	0.9840	0.2090	0.0160
18	0.5529	0.1435	0.4471	0.6737
19	0.6388	0.9336	0.3612	0.0523
20	1.0000	0.9348	0.0000	0.0513
21	0.9833	0.9738	0.0167	0.0206
22	0.9438	0.6205	0.0562	0.2985
23	0.9114	0.4704	0.0886	0.4166
24	0.7333	0.6749	0.2667	0.2558
25	0.6015	0.7853	0.3985	0.1689
26	0.7246	0.5207	0.2754	0.3770
27	0.7041	0.5787	0.2959	0.3314

### Optimization using hybrid Taguchi model

**Figure 4:**
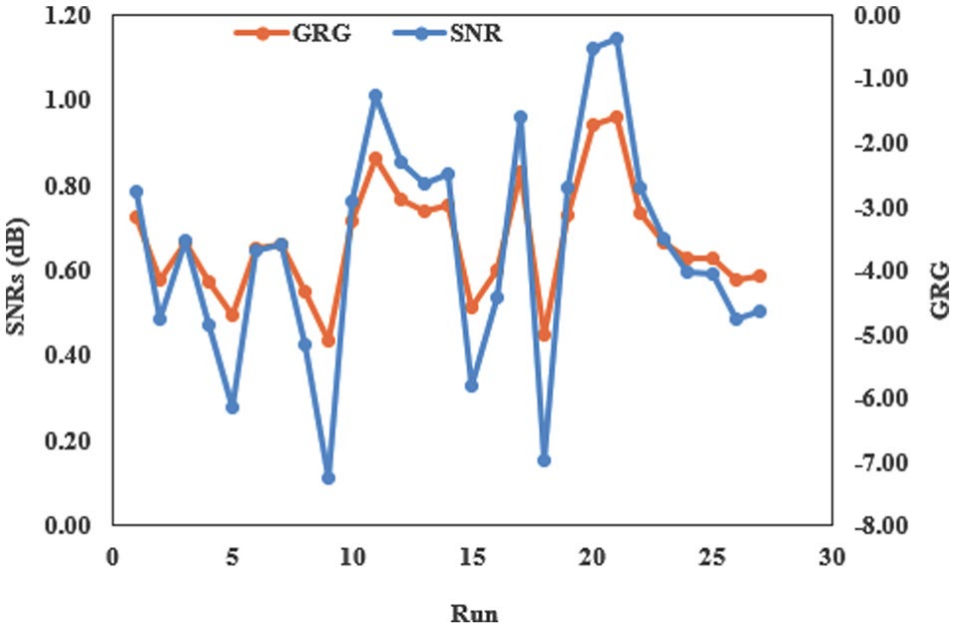
GRG versus SNRs plot.

As seen above, Taguchi can only optimize a parameter at a time and thus it involves more cost, time and effort. Therefore, Deng widely called grey relational analysis (GRA) is principally used to optimize multiple parameters by combining all outputs into an output. Deng is used to unravel real problems made up of a bounded amount of data. It is commonly employed to approximate the properties of indefinite systems having no black and white solution. With respect to grey system black signifies without information whereas white connotes with information. This technique is largely utilized to maximize or minimize problems which have to do with several factors and responses. Data preprocessing through GRA was executed on test data of responses in Table 1 i.e. µ as well as Ks. Table 3 shows the reference sequence obtained by normalization (Eq. 5). In due course, the deviation sequence was computed following Eq. (6) (Table 3). Grey relation coefficient (GRC) and grey relational grade (GRG) of µ as well as Ks were determined using Eqs. (6) and (7), respectively. Subsequently, the mean of GRCs is calculated to establish the GRG. Calculated values of GRGs were employed to produce equivalent SNRs. A larger magnitude of SNR is useful alluding the tests lay in proximity to the actual normalized magnitude of GRG. Figure 4 depicts the plot of GRG against SNRs. It indicates that the 21st trial possesses the highest SNR. Correspondingly, the first rank was designated to 21st trial. The straggling disposition of the GRG, below the plot of SNRs in Fig. 4, also adds to the aforementioned explanation. Ever the ranks determined (Table 5), GRG response table was contrived. Individual factor of GRG at the preferred level was chosen as well as average computed to obtain the mean GRG for separate parameters. The mean response table for the GRG is presented in Table 6.

For example, variable G at level 1 in the first, fourth and seventh runs of the test. The concomitant GRG values in Table 5 were used for computation using Eq. (8). The mean of the chosen GRGs was computed through the method aforementioned put together to generate the mean response table (Table 4). The grades in the response table is used as a degree of correlation^45^. Hence, from Table 4, it is possible to achieve combination of optimum parameters which maximize the overall response. As observed in Table 5, the maximum GRG exists at L3, G1, SD3 and SS3. Therefore, to wrap it up, the best parameter settings for useful abrasive tribological behaviours of filled PTFE composites are load at 9 N, grit size at 1000 mesh, distance at 55 m and sliding speed at 0.14 ms^−1^ coded as L3G1D3S3. ANOVA for GRG shows that grit size with 68.57% ranks as the most influential followed by load with 20.57%, followed by distance having a contribution of 7.78% and finally speed with least contribution of 3.38% for minimum tribological loss. Ramesh and Suresha used Taguchi Deng model to optimize the abrasive wear mode of carbon fabric reinforced epoxy composite filled with Al_2_O_3_ and MoS_2_ as fillers. They reported that optimum parameters for minimum wear rate were found to be load at level 3 (15 N), grit size at level 3 (320), filler content at level 3 (10 wt%) and sliding distance at level 3 (30 m). ANOVA revealed that filler content with 52.08% was the most significant factor affecting the wear mode of the composites^46^.

**Table 4:** Response table for GRGs.

Level	L (N)	G (mesh)	SD ( m)	SS (ms^−1^)
1	0.6218	0.5687	0.6315	0.6665
2	0.6620	0.6651	0.6733	0.6635
3	0.7391	0.7890	0.7181	0.6929
Delta	0.1173	0.2203	0.0867	0.0294
Rank	2	1	3	4

**Table 5:** Rank of GRG with SNRs.

Grey relational coefficient	Grey relational coefficient	Grey relational grade	SNRs(dB)	Rank
µ	Ks			
0.8558	0.5993	0.7276	− 2.7625	10
0.6829	0.4715	0.5772	− 4.7738	21
0.7730	0.5591	0.6660	− 3.5304	13
0.7438	0.4006	0.5722	− 4.8494	22
0.6535	0.3333	0.4934	− 6.1358	25
0.6466	0.6615	0.6541	− 3.6877	15
0.8224	0.5020	0.6622	− 3.5805	14
0.7058	0.3982	0.5520	− 5.1615	23
0.4810	0.3859	0.4335	− 7.2606	27
0.6586	0.7703	0.7145	− 2.9205	11
0.7326	1.0000	0.8663	− 1.2467	3
0.7107	0.8213	0.7660	− 2.3155	5
0.6317	0.8452	0.7384	− 2.6339	7
0.8327	0.6697	0.7512	− 2.4846	6
0.3333	0.6925	0.5129	− 5.7994	24
0.8558	0.3478	0.6018	− 4.4108	18
0.7053	0.9609	0.8331	− 1.5862	4
0.5279	0.3686	0.4483	− 6.9692	26
0.5806	0.8827	0.7316	− 2.7141	9
1.0000	0.8847	0.9424	− 0.5157	2
0.9676	0.9503	0.9589	− 0.3642	1
0.8990	0.5685	0.7338	− 2.6889	8
0.8495	0.4856	0.6676	− 3.5099	12
0.6521	0.6060	0.6290	− 4.0264	16
0.5565	0.6996	0.6280	− 4.0404	17
0.6448	0.5106	0.5777	− 4.7657	20
0.6282	0.5427	0.5855	− 4.6499	19

### Validation

Having determined the optimum parameters, the final phase in Taguchi-Deng is prediction as well as validation of performance enhancement of the dual responses. The predicted GRG was calculated as per Eq. (7). Validation experiments were executed to validate the results of the analysis. Validated results showed that minimum µ and K_s_ were 2.0 × 10^–1^ and 1.5353 × 10^–6^ mm^3^ N^−1^ m^−1^, respectively. More so, it can be implied from Table 6 that the findings of the validation phase are consistent with the computed values. Besides, an enhancement of 55% in GRG was achieved (Table 6). This performance enhancement in the results obtained through the experiments over the initial design parameter confirms the validity of the Taguchi-Deng method for studying the abrasive tribological behaviours of filled PTFE composites. An improvement of 8.4% in GRG was reported by^47^ when the same method was used to optimize wear parameters of silicon nitride reinforced AA6063 matrix composites.

**Table 6:** Results of the confirmatory test.

	Initial design parameter	Optimal parameter	
		Prediction	Validation
Level settings	L1G3SD3SS1	L3G1SD3SS3	L3G1SD3SS3
GRG	0.4335	0.9589	0.9556
Enhancement (%)		54.56	54.66

### Performance appraisal of the models

One of the aims of this work is building hybrid SVR models namely SVR-PSO and SVR-HHO models and compare their efficiency in predicting tribological behaviours of filled PTFE composites. For this objective, tribological behaviours (µ and K_s_) were obtained via experimental results of Table 1. Prediction of tribological behaviours by traditional methods is time and energy consuming due to nonlinearity between tribological independents and dependents of filled polymer composites leading to inaccuracy. These issues can be addressed by nonlinear models. Subsequently, this section details the results achieved in visualized and graphical forms. Before the models simulations, the data was normalized using Eq. (16). Data normalization disallows larger values overshadowing lower values, takes care of units and improves the efficiency of the models.

**Figure 5:**
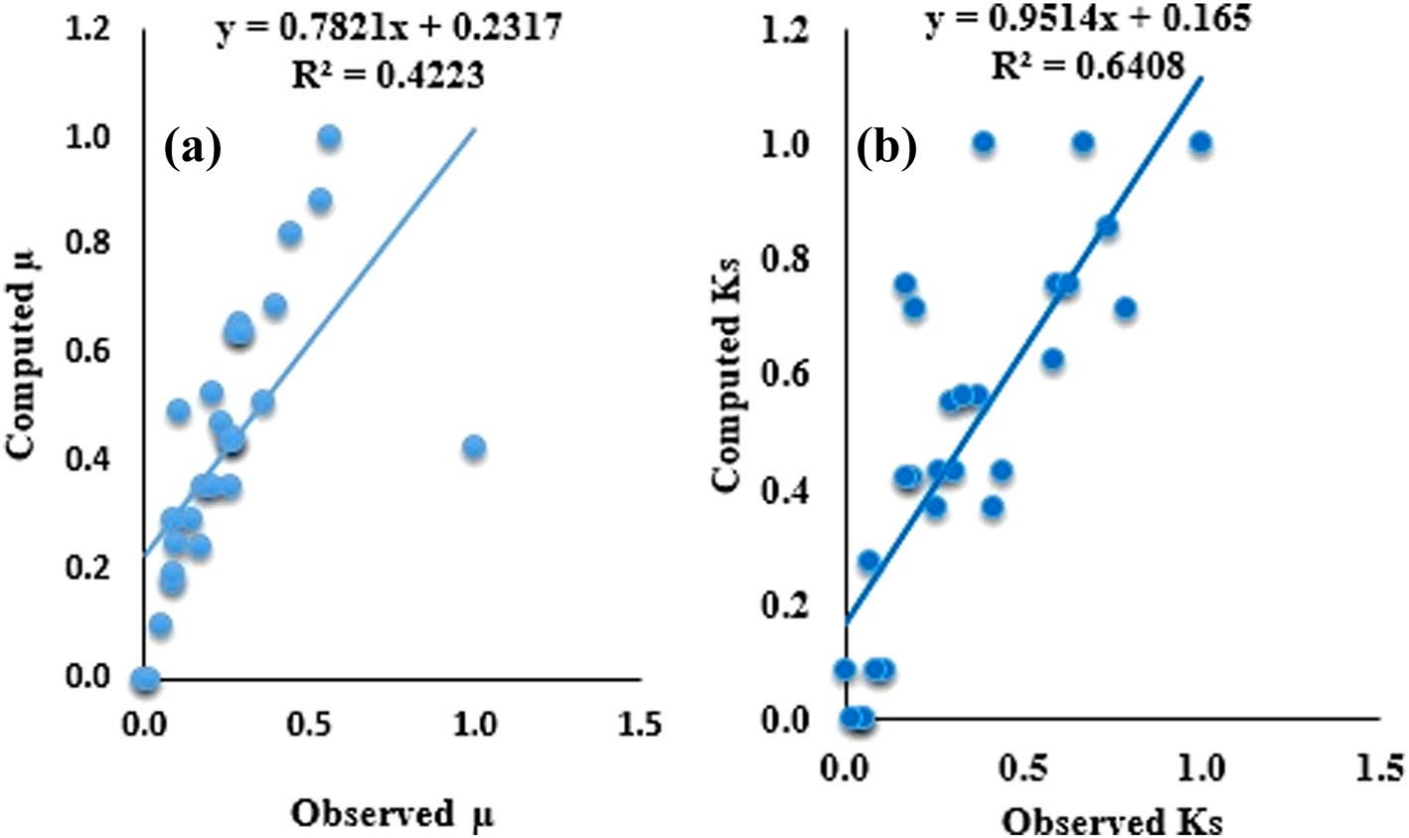
Scatter plot of (**a**) SVR_µ_ model; (**b**) SVR_Ks_ model for the whole data.µ

The simulation process was performed in MATLAB 9.3 (R2020 (a)).Optimized structure of SVR model was chosen via trial-and-error approach. An efficient model is that which allays the prerequisites of model appraisal metrics. Prediction efficiency of the models were appraised using two goodness of fit (R^2^, R) and two prediction error (RMSE, MAPE) metrics in training as well as testing regimes. The simulated outcomes of the individual SVR models for the prediction of µ and K_s_ are quantitatively presented in Table 7. From Table 7, it can be seen that the single SVR models achieved various adequacies according to the statistical evaluation metrics. More so, SVRµ shows best results in terms of goodness of fit in both testing and training stages as compared to SVR_Ks_ model. However, with respect to prediction errors SVR_Ks_ with RMSE 5 × 10^–6^ and MAPE 29% proved to be a relatively adequate model in predicting the tribological behaviours of filled PTFE composites than SVRµ whose accuracy is extremely poor (61%). To have a graphical map of SVR models for the tribological behaviours, a scatter plot is used. A scatter plot gives the degree of agreement between measured and calculated values for the overall goodness of fit. Figure 5a,b depicts the scatter plot of the whole data for SVRµ as well as SVR_Ks_ models_,_ respectively_._ Arising from the scatter plots, it is interesting to note here that SVR_Ks_ model indicated better fitness in comparison to SVR_Ks_ when the whole data points were put together.

**Table 7:** Results of appraisal for single SVR models of µ and K_s._

Models	Calibration						Validation	
	R^2^	R	RMSE	MAPE	R^2^	R	RMSE	MAPE
SVR_Ks_	0.5919	0.7694	0.000005	0.3938	0.5360	0.7321	0.000003	0.2911
SVR_µ_	0.8026	0.8959	0.1974	0.7914	0.8984	0.9478	0.1016	0.6164

**Figure 6:**
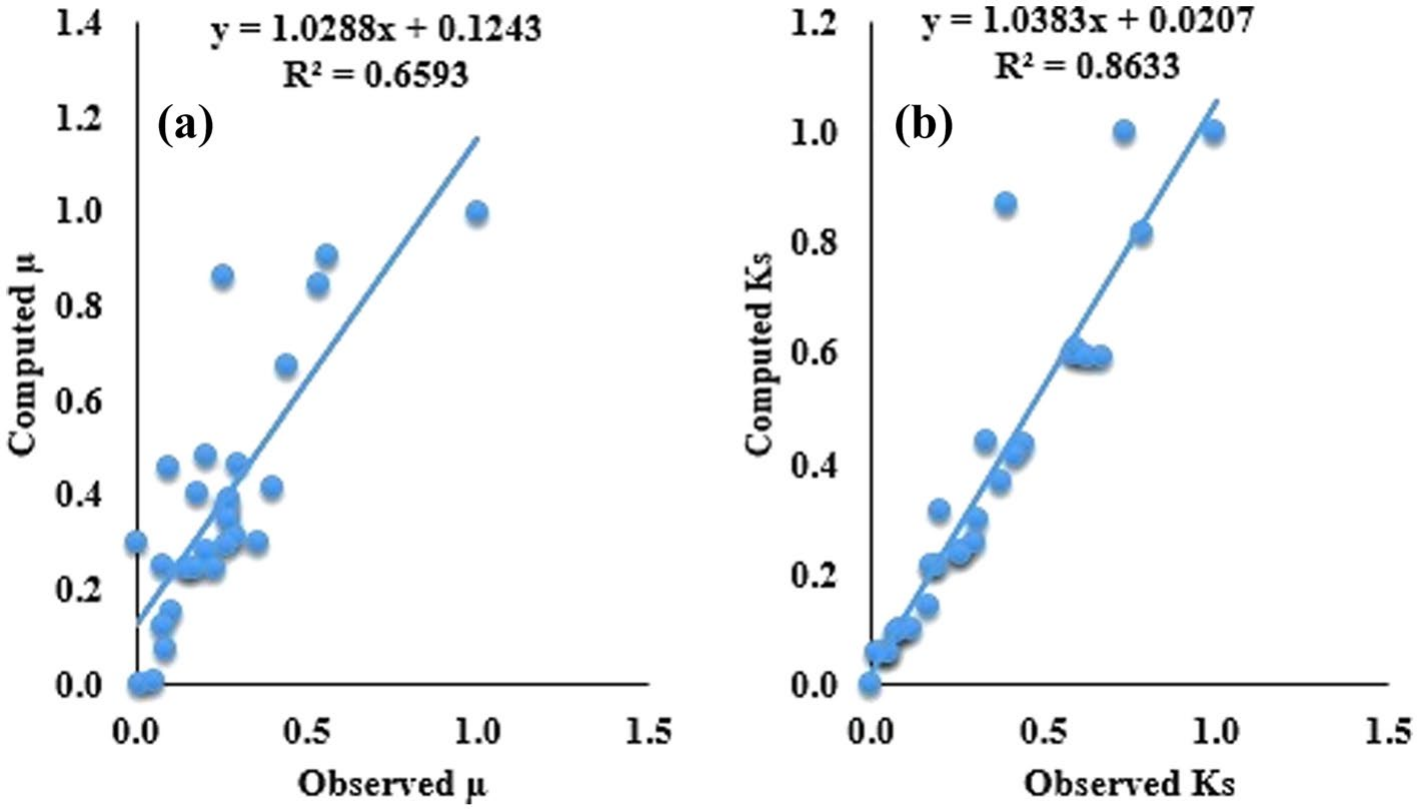
Scatter plot for (**a**) SVR-PSO_µ_ and (**b**) SVR-HHO_Ks_ models all data sets.

**Figure 7:**
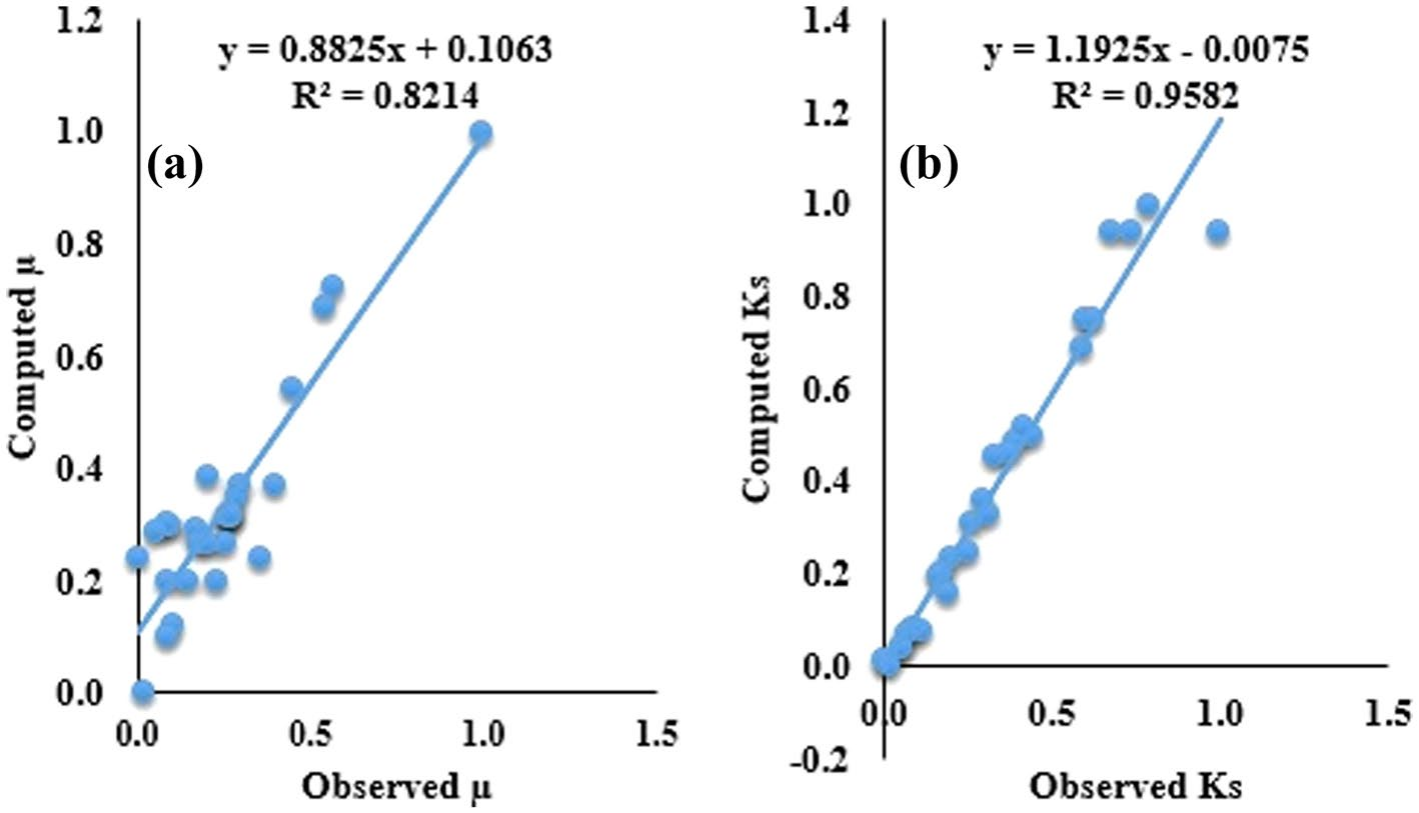
Scatter plot for (**a**) SVR-PSO_µ_ and (**b**) SVR-HHO_Ks_ models for all dataset.

Nevertheless, overall prediction accuracy of the single SVR models was inadequate, especially for SVR_µ_ model. The accuracy can be enhanced using optimization approaches namely PSO and HHO. Fundamentally, it should be considered that the promising prediction accuracy occurred in the course of the training state which is originally used to measure precisely the model based on known inputs and outputs. Nevertheless, the verification stage is significant in appraising the prediction efficiency of the models since it inspects closely the models’ prediction accuracy based upon unknown magnitudes. This advantage is not enjoyed by the training phase. Consequently, a robust model should possess determinate and balanced performance in both training and testing regimes. In general, hybridized models showed a promising ability when compared to un-hybridized models. For consistency the same model evaluation metrics are used to assess the prediction accuracy of the hybridized models. Table 9 shows the results of the hybrid models in both calibration and validation regimes. In spite of the fact it is hard to rank the models as per the model evaluation criteria, the SVR-HHO model indicated higher prediction accuracy in both conditions. From Table 8, it was observed that SVR-HHO_µs_ indicated R^2^ > 90% R = 95%, 99.26%, RMSE > 5%, and MAPE of 5% = Similarly, SVR-HHO_Ks_ R^2^ > 95%, R > 97%, RMSE < 1% as well as MAPE = 3%. This implies SVR-HHO model performed better than SVR-PSO model for prediction of the tribological behaviours of the filled PTFE composites. The predictive superiority of HHO to others is in concord with results obtained by^48^. Figures 6 and 7 present the scatter plot of the SVR hybrid models. Close consistency between measured and calculated points was achieved for SVR-HHO model as compared to SVR-PSO model. More so, R values of the hybrid models lie between 85 and 99%. This agrees with conclusions drawn by^49–51^ that values of R greater than 70% are regarded as acceptable. Therefore, all the optimized hybrid models are acceptable (Table 8).

**Table 8:** Findings of appraisal of the hybrid models for predicting for µ and K_s_.

Models	Calibration				Validation			
	R^2^	R	RMSE	MAPE	R^2^	R	RMSE	MAPE
SVR-PSO_µ_	0.8790	0.9376	0.1210	0.5274	0.9221	0.9603	0.0779	0.0513
SVR-HHO_µ_	0.9123	0.9551	0.0877	0.5139	0.9364	0.9677	0.0636	0.0490
SVR-PSO_Ks_	0.8424	0.9178	0.000003	0.1446	0.9301	0.9644	0.000001	0.1601
SVR-HHO_Ks_	0.9468	0.9730	0.000002	0.0857	0.9853	0.9926	0.000001	0.0322

Zheng et al. used SVR coupled with guided PSO to predict the wear rate of aeroengine. The results indicated better prediction accuracy when compared with single SVR^52^. In a related development, Kahhal et al. optimized and predicted the process parameters of friction stir welding of AH12 1050 using response surface algorithm coupled with PSO model. It was reported that that the hybrid model indicated superior prediction accuracy^53^. The prediction efficiency of HHO coupled with generalized neural network was reported in the literature^54^ to predict the abrasion resistance of ultra-strength martensitic steel. The results showed that observed and the computed values of the wear property lie within the uncertainty range of 3–4%. In a dissimilar fashion, Sammen et al. hybridized artificial neural network (ANN) with PSO, HHO and genetic algorithm (GA) to predict the scour depth downstream of ski-jump spillway. It was observed that of all the hybrid models, ANN-HHO model superior prediction accuracy with mean absolute error of 0.1760 and root mean square error of 0.2538^54^. The findings in these literatures reinforced the results of this study that hybrid models could increase the effectiveness of single models.

### Comparing the performance of the models

**Figure 8:**
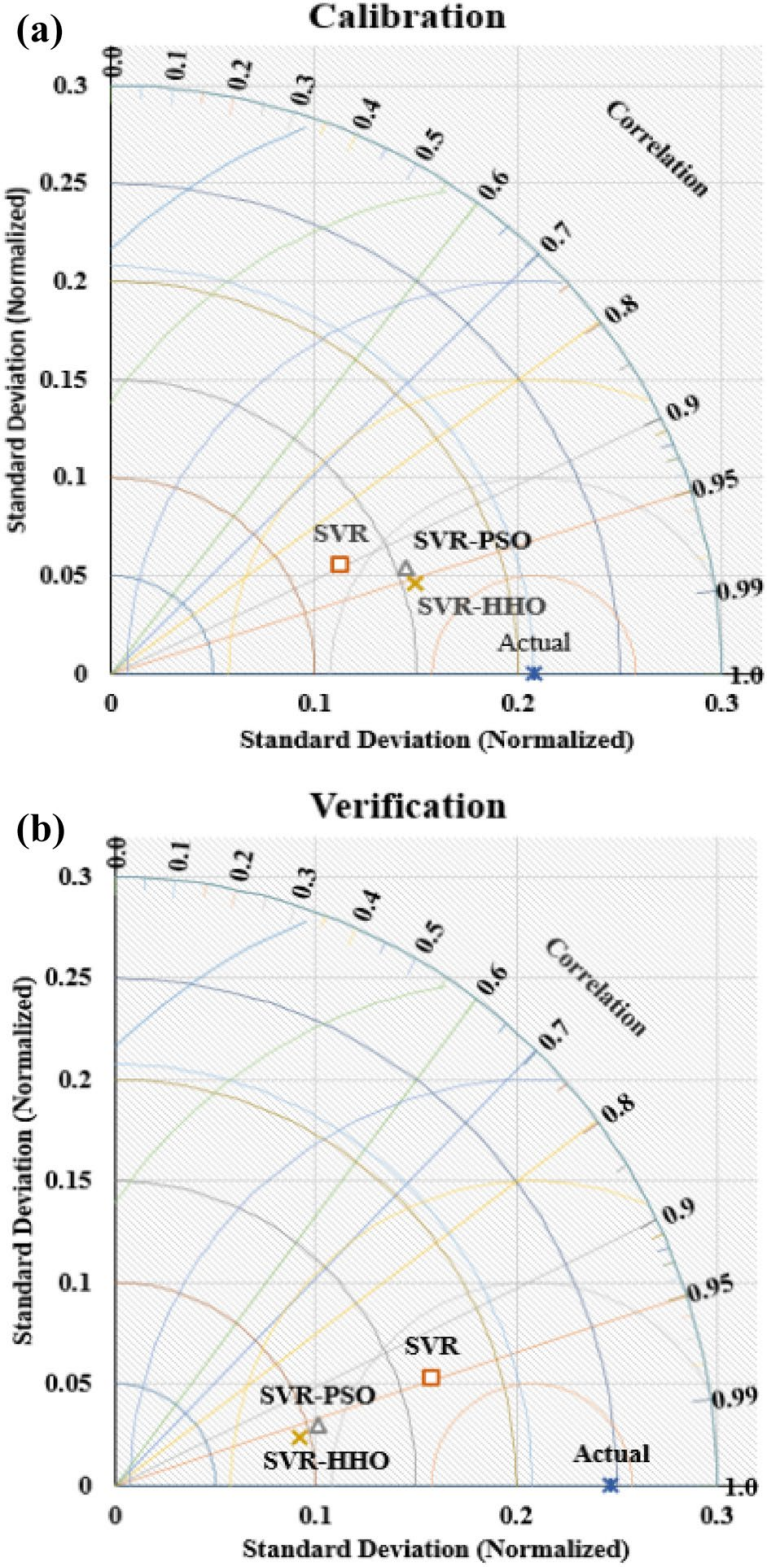
Taylor diagram for µ models in (**a**) calibration stage and (**b**) verification stages.

**Figure 9:**
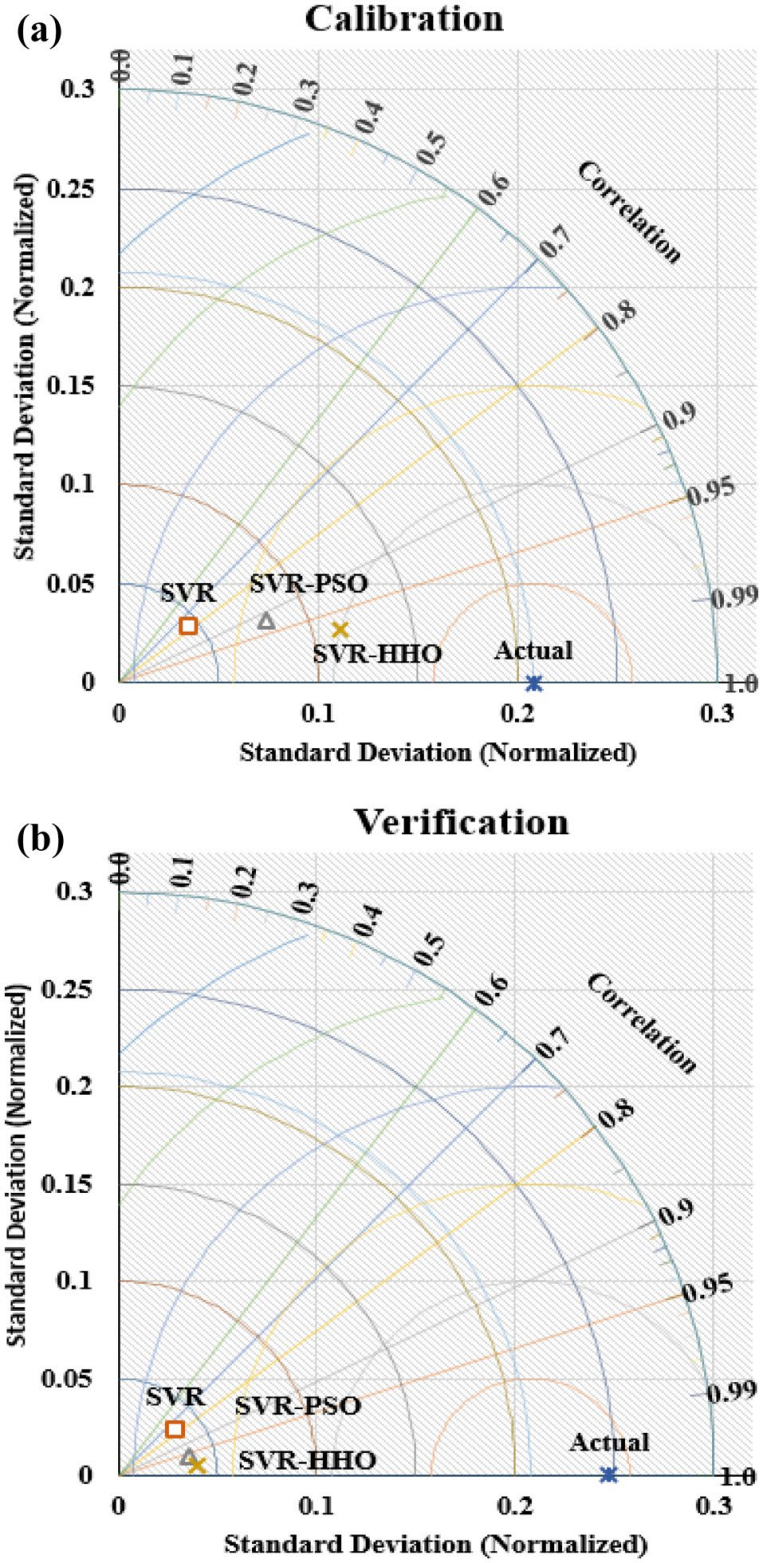
Taylor diagram for K_s_ models in (**a**) calibration and (**b**) verification of the models.

**Figure 10:**
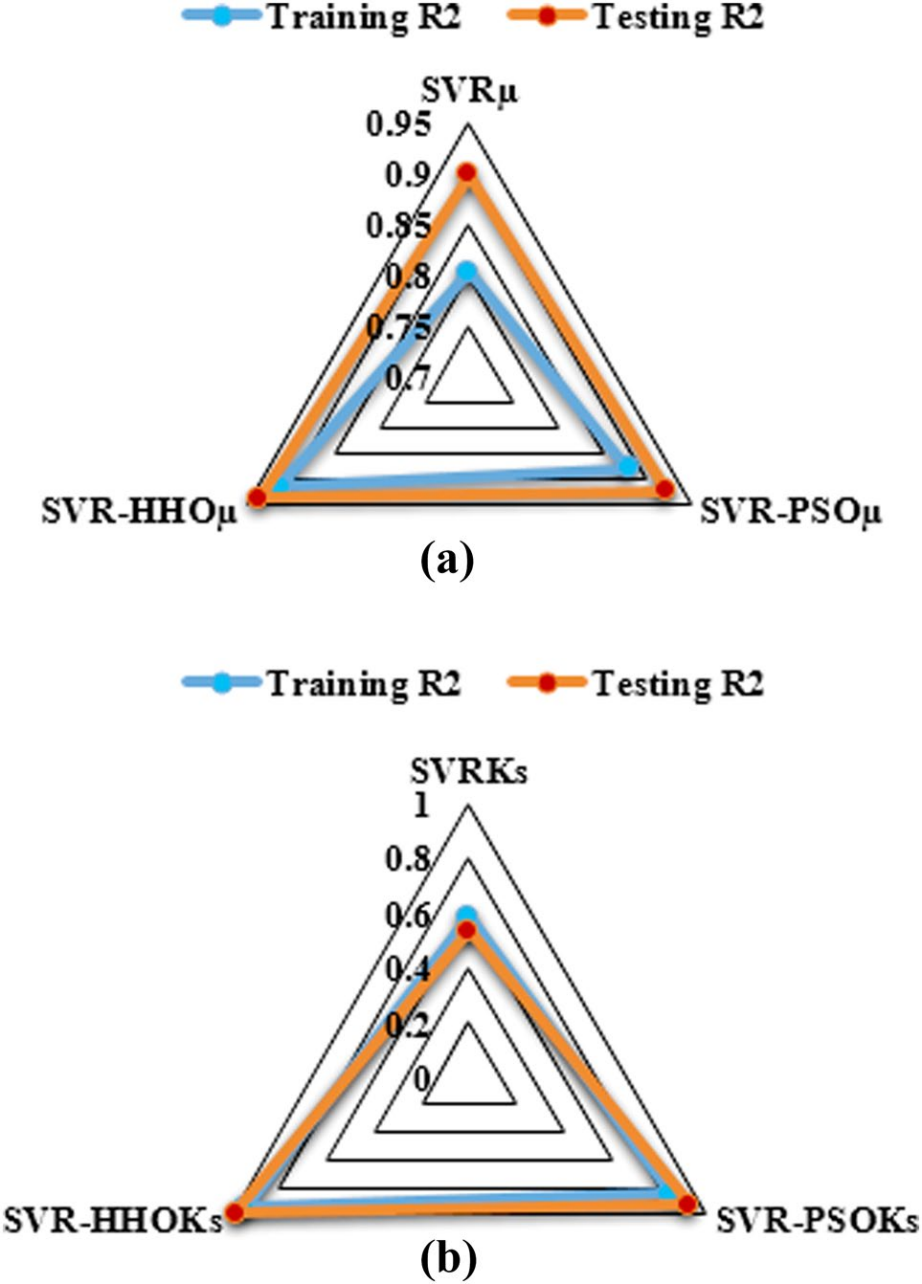
Radar plot for (**a**) µ and (**b**) Ks in calibration and verification regimes.

The SVR model and its hybrids namely SVR-PSO and SVR-HHO models are compared via 2D Taylor’s plot as shown in Figs. 8 and 9, respectively. As seen in the Taylor’s plot SVR-HHO model indicated better fitness in both cases with values of 97% and 99% for µ and Ks, respectively in the calibration regime. Therefore, it can wrapped up that SVR, SVR-PSO and SVR-HHO models can understand and follow the intricate and nonlinear correlation between tribological input parameters and response parameters of filled PTFE composites in abrasive conditions. Additional analysis can be done using a radar plot for the prediction of the µ and Ks as shown in Fig. 10. It can as well be seen that SVR-HHO_µ_ > SVR-PSO_µ_ > SVR_µ_ and SVR-HHO_Ks_ > SVR-PSO_Ks_ > SVR_Ks_. This implies that in both cases SVR-HHO model was able of capturing the best fitting trend of the tribological behaviours of filled PTFE composites.

In spite of the availability of ample of predictive models, there is no particular model that can guarantee consistent optimal performance in addressing various types of issues. Yet, latest research on unique population-based as well as nature inspired optimization paradigm (HHO) models indicated better adequacy in establishing optimal solutions for multi-objective problems. The statistical analysis outcomes and comparisons revealed that SVR-HHO model yields promising and often competitive outcomes than well-established models.

Multi linear regression MLR has been widely used as conventional approach for prediction tribological behaviour of materials. Ikpambese and Lawrence used MLR and artificial neural network (ANN) to predict friction and tribological rate of reinforced palm kernel epoxy for brake pad application. The prediction accuracy in terms of RMSE for the MLR and ANN models were found to be 0.0082 and 0.00450, respectively^55^. These values are less effective compared to RMSE of 0.000001 for SVR-PSO and SVR-HHO in this article. Similarly, Altay et al. used LR, SVM and Gaussian process regression to predict the wear rate of ferro-alloy coating^56^. They reported RMSE and R^2^ for LR, SVM and GPR as 0.86, 0.69, 0.69 and 0.93, 0.96 and 0.96, respectively which were higher than the reported RMSE and R^2^ of this article was higher than as depicted in Table 8. With this it can be said that the proposed models of this study are more effective than the conventional models in predicting the wear rate of materials.

Using the proposed models in the scientific community and industry for the optimization and prediction of composite materials will lead to many benefits. Among these benefits are significant aspects such as minimization of cost, reduction in human effort, prevention of time loss during experiments. The developed models are expected to be specifically applied in the design and/or modification and development of existing/new materials for tribological applications in agricultural machinery, rolling industry, and mining industry, such as crushing and milling.

## Conclusions

In this study, multi-response optimization and prediction of objective functions of coefficient of friction (µ) and specific wear rate (K_s_) of tribological behaviour of filled polytetrafluoroethylene (PTFE) composites was presented using Taguchi Deng and two hybrid SVR models (SVR-PSO and SVR-HHO). More so, effect of load, grit size, distance and speed on the two responses were studied. It was found that as load, grit size and speed increased µ and Ks decreased. However, increase in distance led to increase in µ and decrease in Ks. Based on the Taguchi Deng optimization, the optimum parameter levels were found to be load at level 3 (9 N), grit size at level 1 (1000 mesh), distance at level 3 (55 m) and speed at level 3 (0.14 ms^−1^) coded as L3G1SD3SS3. ANOVA for GRGs indicated that grit size with 68.57% was the most influential parameter, followed by load with 20.57%, followed by distance with 7.78% and then speed with least influence of 3.38% affecting the tribological behaviours of filled PTFE composites. Validation test revealed that there was an enhancement of 55% in GRG from 0.4335(L1G3SD3SS1) for initial design settings to 0.9589 for the optimized levels (L3G1SD3SS3). For the prediction accuracy of the models, it was found that SVR_Ks_ outperformed SVR_µ_ model. As regards to hybrid models, there was an increase in prediction effectiveness for both SVR-PSO and SVR-HHO over SVR. Even though both SVR-HHO and SVR-PSO models were able to accurately predict the µ and Ks, SVR-HHO model exhibited the lowest prediction error of 4.06% on the average as compared to SVR-PSO model whose prediction accuracy was found to be 10.57% on the average. The integration of Taguchi with Deng approach and SVR with PSO and HHO resulted in the optimization and prediction of tribological behaviour of reinforced PTFE composites with low experimental cost and superior accuracy.

## Methods

### Experimental set up

**Figure 11:**
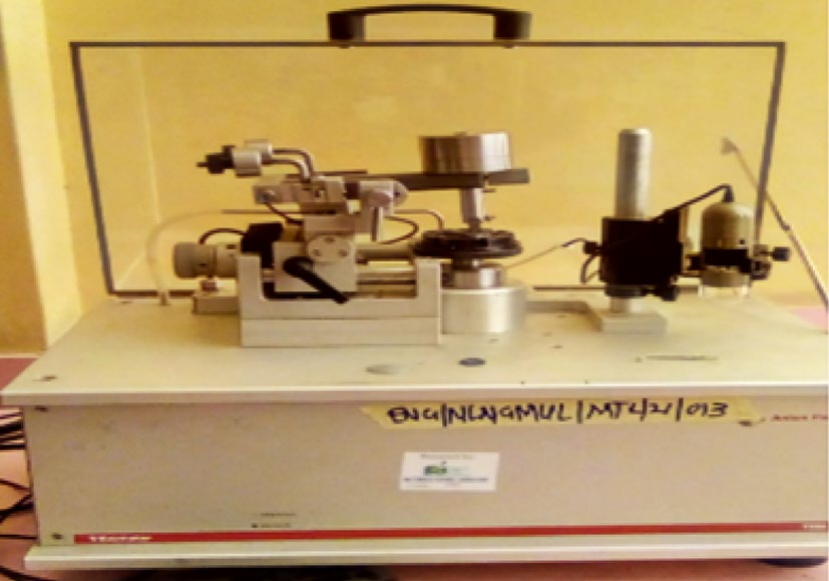
Arton Paar Tribometer used for the experiment.

The materials utilized in this work are polytetrafluoroethylene (PTFE), carbon-filled composites (CF25) and bronze-filled composites (BF40) because of their availability and wider applications. Abrasive tribological experiment was conducted according to ASTM G99 standard using pin-on-disc tribometer (Model: Arton Paar, Made in Switzerland) shown in Fig. 11. The counterface material for the wear test is a steel of disc 140 mm in diameter and thickness of 10 mm that has been heat treated to obtain a surface hardness of 55–60 RC. This is grounded to a surface finish of nearly 0.12 µm centerline average. The samples of dimension 20 mm long, width 10 mm and depth 6 mm were cut from compression moulded rectangular plates whose dimensions are (500 × 500 × 6) mm using computer numerical control water jet machining for the pin-on-disc abrasive experiment. A specially designed fixture for holding the square samples was designed and fabricated. The samples were inserted into the fixture, bolted and then loaded against silicon carbide (SiC) abrasive papers glued to the hardened steel holder by means of liquid adhesive. Control parameters and their levels are shown in Table 9.The experimental design is as shown in Table 10. In all the experiments, mass before (m1) and mass after (m2) was measured using digital weighing balance (Model: PS 1000.RS RADWAG, Made in Poland) with 10–3 g precision accuracy. Test was performed at room temperature (29 °C and relative humidity 55%). Samples were cleaned with a brush before and after the experiment to get rid of debris and then weighed. The loss in pin weight (WL), volume (VL) and specific wear rake (Ks) was determined through the mathematical Eqs. (1), (2) and (3), respectively.

1 $$\mathrm{WL}={m}_{b}-{m}_{a}$$

2 $$\mathrm{VL}= \frac{{\mathrm{M}}_{\mathrm{L}}}{\uprho }$$

3 $${\mathrm{K}}_{\mathrm{S}}= \frac{{\mathrm{M}}_{\mathrm{L}}}\rho{\mathrm{ LD}}$$

where ${\mathrm{W}}_{\mathrm{L}}$= weight loss (g), ${\mathrm{V}}_{\mathrm{L}}$ = volume loss (mm^3^) ${m}_{b}$= mass before test (g), ${m}_{a}$= mass after test (g), $\uprho$ = (g cm^−3^) of materials, L = load in N and D = sliding distance (m). Each trial was performed twice and averaged.

**Table 9:** Parameters and their levels.

Parameters	Symbol	Level 1	Level 2	Level 3
Load, (N)	L	3	6	9
Grit, (mesh)	G	1000	400	150
Sliding distance, (m)	SD	25	45	55
Sliding speed , (ms^−1^)	SS	0.04	0.08	0.14

**Table 10:** Experimental design of reinforced PTFE composites against SiC particle based on Taguchi ${L}_{27}$(3^4^) OA.

Trial	L (N)	G (mesh)	SD (m)	SS (ms^−1^)
1	3	1000	25	0.04
2	3	1000	25	0.04
3	3	1000	25	0.04
4	3	400	45	0.08
5	3	400	45	0.08
6	3	400	45	0.08
7	3	150	55	0.14
8	3	150	55	0.14
9	3	150	55	0.14
10	6	1000	45	0.14
11	6	1000	45	0.14
12	6	1000	45	0.14
13	6	400	55	0.04
14	6	400	55	0.04
15	9	1000	55	0.14
16	6	150	25	0.08
17	6	150	25	0.08
18	6	150	25	0.08
19	9	1000	55	0.08
20	9	1000	55	0.08
21	9	1000	55	0.08
22	9	400	25	0.14
23	9	400	25	0.14
24	9	400	25	0.14
25	9	150	45	0.04
26	9	150	45	0.04
27	9	150	45	0.04

### Taguchi optimization approach

Single parameter optimization and percentage contribution of each parameter can be performed using Taguchi L_27_ (34) orthogonal array (OA). Taguchi is an optimization process to establish the best process parameter. In this study, four parameters with three levels (3^4^) are considered for the configuration of the test. Twenty seven trials have been performed based on Taguchi L_27_ (3^4^) OA as shown in Table 10. The Taguchi method was chosen in this study because of its simplicity of analysis, substantial reduction in the cost of experiment and validity over a wide region spanned by the control factors and their settings. Signal to noise ratios (SNRs) are used to establish the optimum parameters. There exist three types of quality loss function for SNRs namely: nominal the better; higher is the better; lower the better. Thus, the lower the better function was used to obtain the optimum wear-rate parameters in this study. SNRs for µ and Ks was computed using Eq. (4) for all the 27 trials in accordance with Taguchi ${L}_{27}$(3^4^) OA.

4 $$\left(\mathrm{SNR}\right)\mathrm{STB}=-\mathrm{log}10\frac{1}{\mathrm{n}}(\sum_{\mathrm{i}=0}^{\mathrm{n}}{\left({\mathrm{y}}_{i}\right)}^{2})$$

where n = number of experiments and ${\mathrm{y}}_{i}$ = experimental value.

### Optimization using Taguchi-Deng approach

Taguchi optimization is capable only of optimizing a single response. However, when two or more responses of distinct features are involved Taguchi technique is limited. Thus an optimization method called Deng popularly referred to as grey relational analysis (GRA) becomes a panacea. This approach is capable of addressing the limitation imposed by Taguchi approach by integrating multiple responses into a single response whose optimal parameters combination represents the several responses thereby minimizing cost and time spent while using Taguchi optimization method. Taguchi L_27_ (4^3^) OA with Deng was used to obtain the optimum levels of tribological parameters. Data normalization is categorized as smaller or larger the better. Let the actual sequence and the comparison sequences be ${X}_{i}^{*}\left(k\right)$ and ${\varphi }_{i}\left(k\right)$, respectively. $\mathrm{i}$ = 1, 2, 3….; m = 1, 2, 3…and n and m represent the total number of experiments and experimental values, respectively. Data preprocessing is used to transform the actual sequence into an identical sequence. Many data preprocessing techniques can be utilized in Taguchi-Deng method, depending upon the features of the actual sequence. Generally, series is normalized between 0 and 1^20^. For this study, the target value is “the smaller the better”. Consequently, the actual sequence is pre-processed via Eq. (5).

5 $${X}_{i}^{*}\left(k\right)=\frac{max{\varphi }_{i}\left(k\right)-{\varphi }_{i}\left(k\right)}{max{\varphi }_{i}\left(k\right)-{min\varphi }_{i}\left(k\right)}$$

where ${X}_{i}^{*}\left(k\right)$ = normalized for the ith experiment and ${\varphi }_{i}\left(k\right)$ = initial sequence of the average responses. After data normalization, the succeeding phase is computation of deviation sequence of the normalized data using Eq. (6).

6 $$\Delta _{{{\text{oi}}}} \left( {\text{k}} \right) = {{ |}}_{0}^{*} \left( {\text{k}} \right) - {\text{X}}_{{\text{i}}}^{*} \left( {\text{k}} \right)|$$

where ${\Delta }_{oi}\left(k\right)$ = deviation, ${X}_{0}^{*}\left(k\right)$ = normalized data and ${X}_{i}^{*}\left(k\right)$ = comparability sequence. Grey relational coefficient (GRC) is thus estimated through Eq. (7).

7 $${\upxi }_{\mathrm{i}}\left(\mathrm{k}\right)= \frac{{\Delta }_{\mathrm{min}}+{\upzeta \Delta }_{\mathrm{max}}}{{\Delta }_{\mathrm{oi}}\left(\mathrm{k}\right)+{\upzeta \Delta }_{\mathrm{max}}}$$

where ${\xi }_{i}\left(k\right)$= GRC of each response, ${\Delta }_{min}$ and ${\Delta }_{max}$ = lowest and the highest deviations of the individual target factor, respectively. Differentiating or identification coefficient is symbolized by $\zeta$ and is demarcated within the range of $\zeta \epsilon \left[\mathrm{0,1}\right]$. This is usually set at ½ to assign equivalent weights to every variable. As indicated in (Eq. 8) GRG is then determined by taking mean of GRG of each output parameter:

8 $${\gamma }_{i}= \frac{1}{n}\sum_{i=1}^{n}{\xi }_{i}(k)$$

where ${\gamma }_{i}$ = GRG obtained for ith test run, n = summation count of performance attributes. Following the determination of the optimal levels of parameter, the last phase is to predict and validate the result using Eq. (9):

9 $${\gamma }_{predicted}= {\gamma }_{m}+\sum_{i=1}^{q}{\gamma }_{0}-{\gamma }_{m}$$

where ${\gamma }_{0}$ represents the highest of mean GRG at optimum levels of variables and ${\gamma }_{m}$ defines the average GRG. $q$ = parameter that signifies factors influencing the target values.

### Support vector regression (SVR) model

In 1995 Vapnik contrived and implemented support vector machine (SVM) was contrived and implemented, which is regarded an observer-based learning approach. The minimization of structural risk as well as statistical learning theory are the most important function of the SVM. Nevertheless, the properties which distinguish SVM from ANN are complexities, minimization of error as well as gain in the network’s performance capability. SVM can be categorized into linear support regression as well as nonlinear support regression (NSVR). Several engineering fields such as have witnessed the application of SVM’s kernel function. SVR model could be thought of as SVM on the basis of layers which include kernel function weighting on the inputs as well as function weighted sum of kernel targets. By and large, SVM is codified into two codes namely Support Vector Regression (SVR) and Support Vector Classifier (SVC) models. SVR model is made up of predictions whereas SVC model treats classifications. SVR model is designated as:

10 $$f\left(x\right) = w\times\Phi (x)+ b$$

where w stands for weight of the vector displayed in feature space, $\Phi$ shows the transfer function, b is bias. Therefore, in order to show the SVR function $f\left(x\right)$, problem of regression is presented as:

11 $$\mathrm{Minimize}:\frac{\parallel w{\parallel }^{2}}{2}+ C\left[ {\sum }_{i=1}^{N} \xi + {\xi }^{*}\right]$$

Subject to the conditions:

11a $${y}_{i}-f\left(x\right)\le + \varepsilon +{\xi }_{i}$$

11b $$f\left(x\right)-{y}_{i}\le + \varepsilon + {\xi }^{*}$$

11c $${\xi }_{i}, {{\xi }^{*}}_{i} \ge 0, i=\mathrm{1,2},\dots N$$

where $\parallel w{\parallel }^{2}$ = weight norm vector, C = penalty parameter, ${\xi }_{i}$ and ${\xi }^{*}$= slack variables. By using Lagrange functions, the solution of the nonlinear regression function can be presented based on optimization as follows:

12 $$f\left(x\right)= \sum_{i=1}^{N}\left({\alpha }_{i }- {\alpha }_{i}^{*}\right)K\left(x,{x}_{i}\right)+b$$

where $K\left(x,{x}_{i}\right)$ shows the kernel function and are binary variables (${\alpha }_{i }and {\alpha }_{i}^{*}>0)$. There exist several kinds of kernel functions including sigmoid, linear, polynomial but the commonly used kernel function is the radial basis function (RBF). Consequently, the RBF kernel was used in this study and it is expressed as (Eq. 13).

13 $$K\left(x,{x}_{i}\right)=exp(-\gamma \parallel {x}_{i}-x{\parallel }^{2})$$

where $\gamma$ = kernel parameter. SVR model performance is affected by C, $\gamma$ and $\varepsilon$ (size).

### Harris Hawk optimization (HHO) model

**Figure 12:**
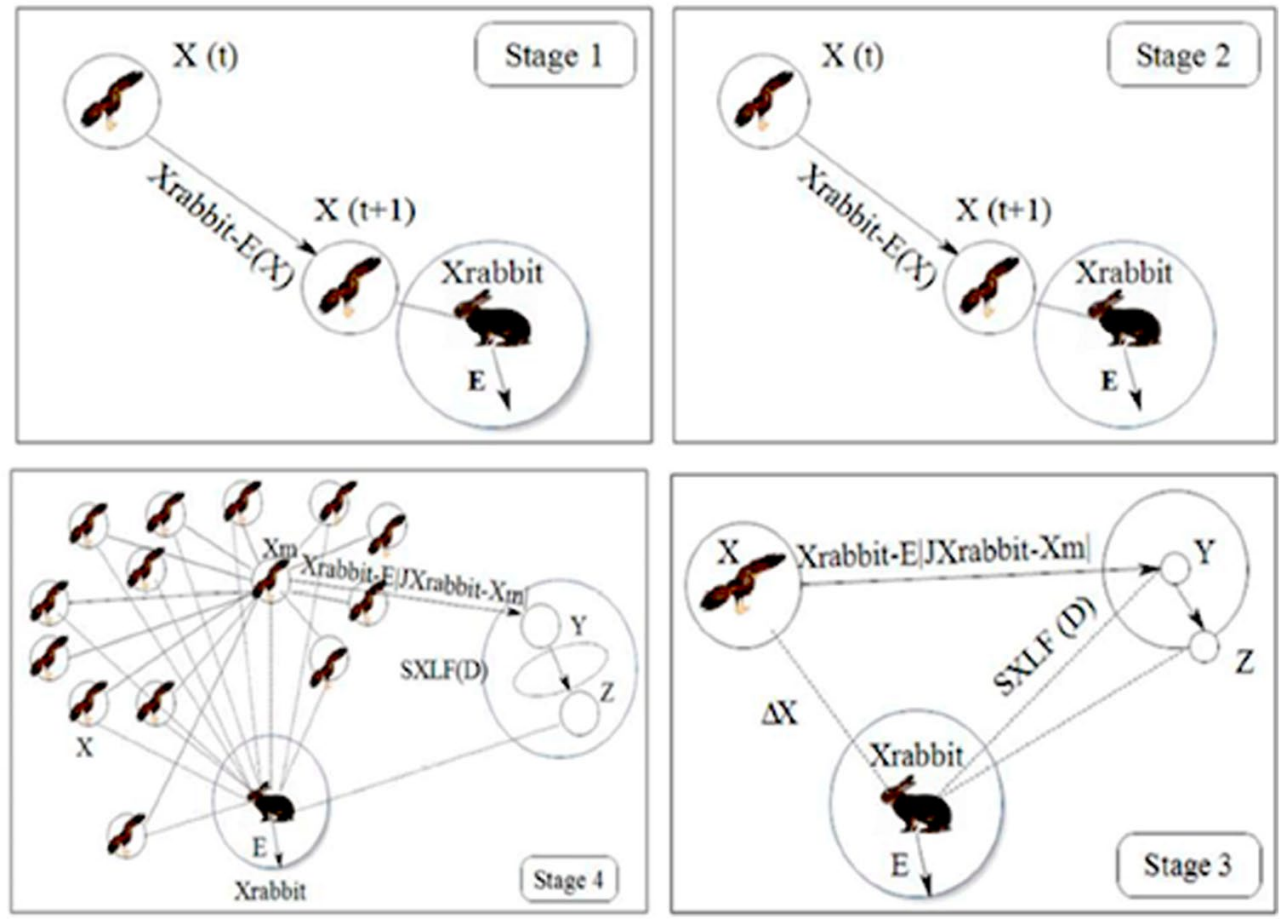
Various stages in HHO^57^.

HHO is a unique model worked out by simulating the hawk’s hunting process. Lately, the procedure has been used with success in solving several intricate engineering as well as science issues. The hawks mostly operate alone whereas the Harris hawks pursue and hunt through operating and cooperating together. Hence, the HHO method is similar to Harris Hawks’ natural hunting characteristic and cooperative methodology. HHO model hunting methodology entails tracing, encircling, approaching and attacking. These mechanisms are achieved in three principal phases namely: exploration, a transition from exploration to exploitation as well as exploitation (Fig. 12).

### Particle swarm optimization (PSO) model

**Figure 13:**
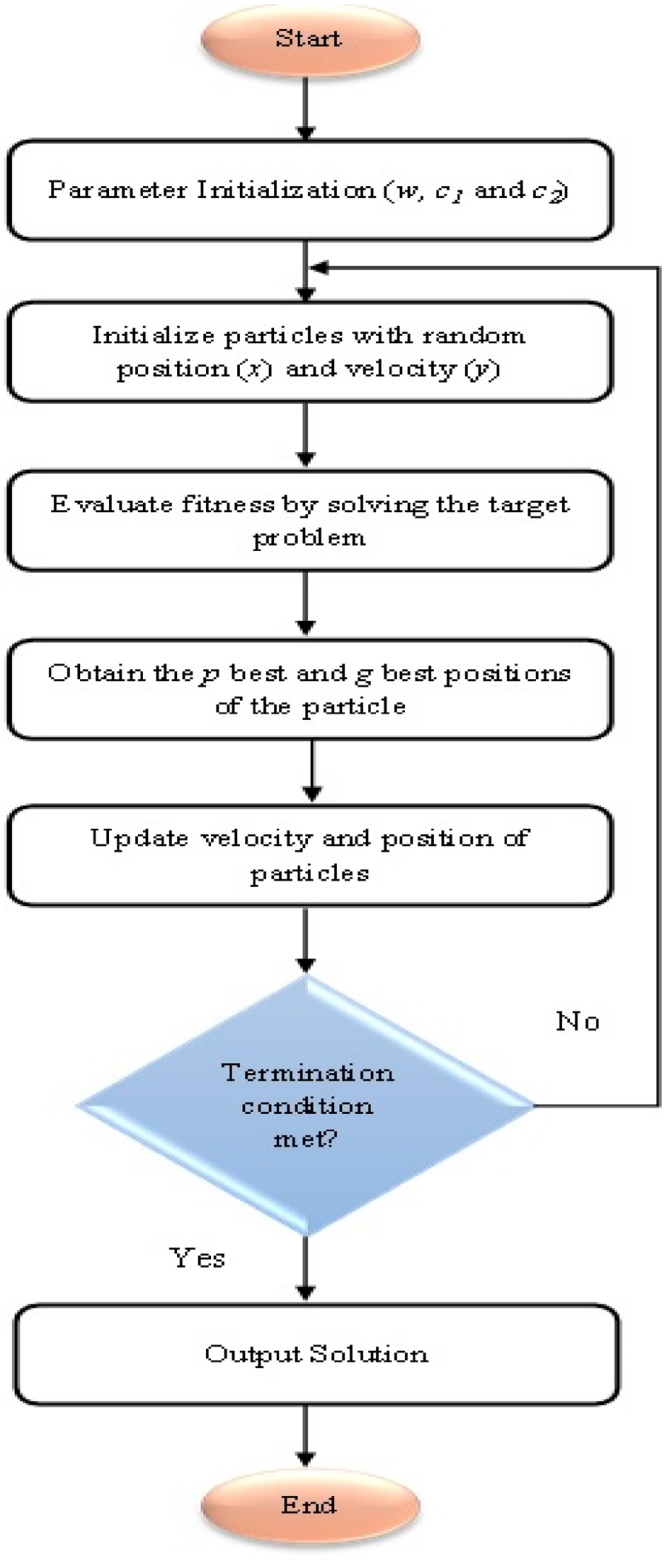
PSO algorithm flow chart.

In 1995, PSO was presented by Kennedy and Eberhart. It is a search method based on population which is inspired by the social behaviour and dynamics of animals. The initial intention of the SPO philosophy was to clearly mimic animals’ social behaviour bird flocking as an instance to detect trends that control capability of birds to fly with precision at the same time and to all of a sudden alter the direction with regathering in an optimum style. Arising from this first purpose, the philosophy inspired into a simple and efficient optimization approach. PSO is initiated with a group of random particles that look into an optimum value by updating the two best values in each iteration. The first one is named the personal best (pbest). This is the best value so far obtained by any particle in the population. All the particles explore the search space and the information collected by them is utilized for finding the best particle in the swarm referred to as global best (gbest). Thereafter, the particle updates its velocity and positions according to Eqs. (14, 15):

14 $${V}_{i}^{k+1}= \omega {V}_{i}^{k}+ {c}_{1}{r}_{1}\times \left({pbest}_{i}^{k}-{X}_{i}^{k}\right)+ {c}_{2}{r}_{1}\times ({gbest}^{k}-{X}_{i}^{k})$$

15 $${X}_{i}^{k+1}={X}_{i}^{k}+{V}_{i}^{k+1}$$

where ${V}_{i}^{k+1}$= the velocity of individual I at iteration $k$ +1, ${V}_{i}^{k}$ = the velocity of individual i at iteration $k$, $\omega$ stands for inertia weight parameter, ${c}_{1}$ and ${c}_{2}$ show the cognitive parameters, ${r}_{1}$ and ${r}_{2}$ = random numbers between 0 and 1, ${X}_{i}^{k}$ = position of individual i at iteration k, ${pbest}_{i}^{k}$ = the best position of individual I at iteration k and ${gbest}^{k}$ indicates the best position of the group until iteration k. Figure 13 shows the flowchart of the PSO algorithm.

### Hybrid SVR model

**Figure 14:**
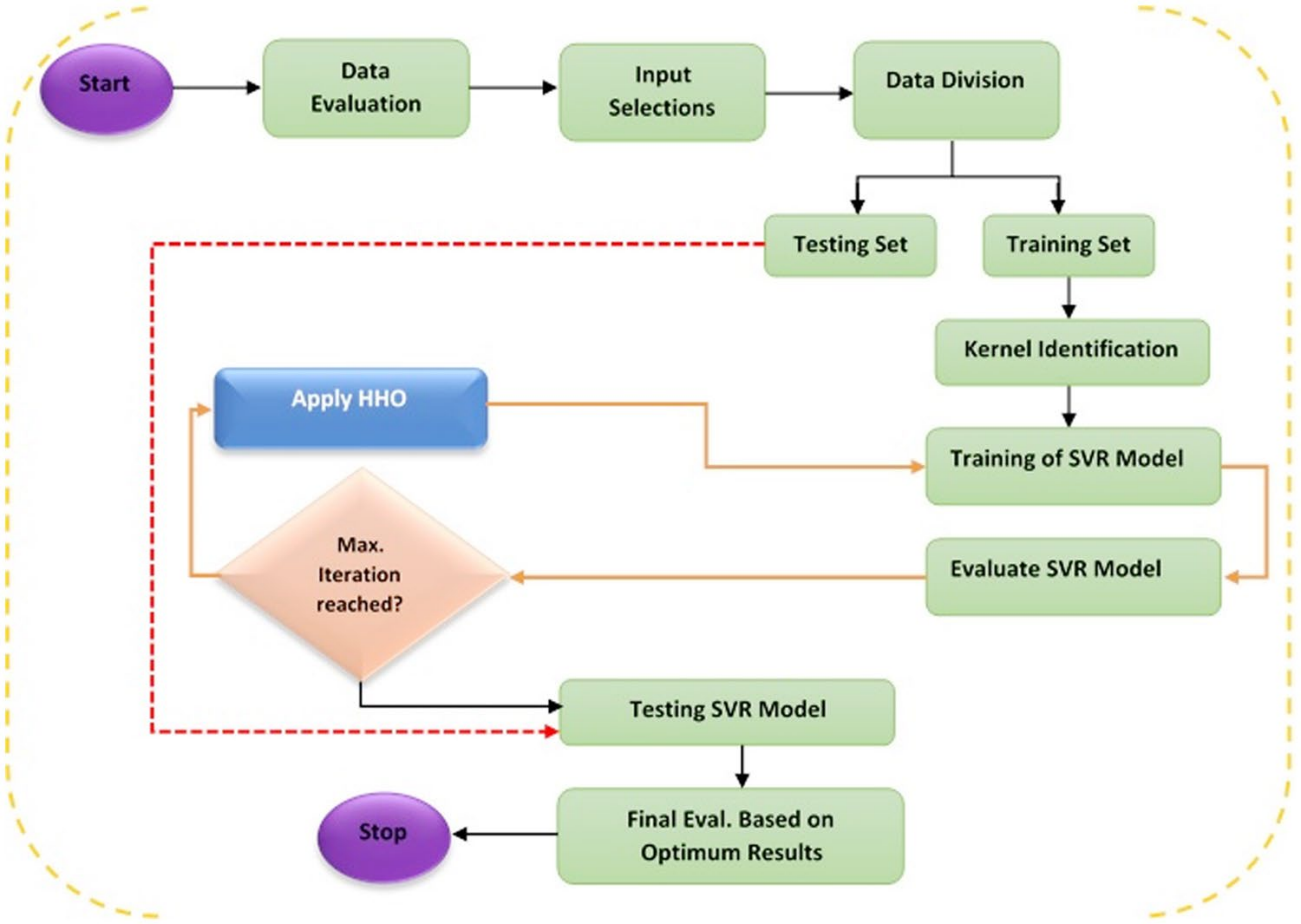
Flow chart for the proposed hybrid models.

Improving the SVR model’s performance needs a cautious delineation of parameters involved in the SVR model. The strength of the SVR model relies upon the precise choice of C, $\gamma$ and $\varepsilon$. Yet, these parameters having a wide range make the search space very large thus making it difficult to choose precise parameters. Therefore, this issue can be addressed as optimization issue that requires sorting out via optimization methods. Integration of SVR model with PSO as well as HHO models that are algorithms inspired by nature led to the following hybrid model namely: SVR-PSO and SVR-HHO for the prediction of tribological behaviours of filled PTFE composites. The nature inspired models were utilized to choose the SVR model parameters viz: C, $\gamma$ and $\varepsilon$. Proposed flow-chart of the hybrid model illustrated in Fig. 14.

### Data pre-processing, model validation and performance metrics

One of the significant aims of any soft computing model is to ascertain that models conform to acceptable data based on the models evaluation metrics utilized to obtain a dependable and strong computed outcome of the unknown data. Nevertheless, overfitting as well as local minima problems occur in the data validation. Hence, the performance of the learning phase might be unsatisfactory. This is especially when the analysis deals with a relatively small amount of dataset, as in this study. Various validation methods can be employed including cross-validation (k-fold), hide-out and leave one out. Here, the k-fold approach was used to repeal overfitting issues. With respect to this study, the data was split into (70%) and (30%) for training and testing, respectively. The data obtained through abrasive experiments was pre-processed and normalized according to Eq. (16). Data normalization was performed prior to model training and it usually enhances the efficiency of the predictive models. The current work introduced SVR model coupled with particle swarm optimization (PSO) and Harris Hawk optimization (HHO) models to predict abrasive tribological behaviours of filled PTFE composites. Prediction of tribological behaviours is important. However, creation of a reliable model is often challenging and difficult given the nature of the data set obtained from the experiments.

16 $$y=\left(\frac{x-{x}_{min}}{{x}_{max}-{x}_{min}}\right)$$

where y = normalized data, $x$ = is the experimental data while ${x}_{max} and {x}_{min}$ are the maximum and minimum experimental data, respectively.

Generally, model efficiency performance should include at least one goodness of fit and at least one prediction error metrics^58^. Based on this determination coefficient (R^2^), correlation coefficient (R), root mean square error (RMSE) and mean absolute percentage error (MAPE) are chosen as models appraisal metrics of the soft computing methods. R^2^, R, RMSE and MAPE are given below. These statistical tools furnish the information on efficiency of models.

17 $${R}^{2}=1-\frac{\sum_{i-=1}^{N}({x-y)}^{2}}{\sum_{i=1}^{N}{(x-\widehat{x})}^{2}}$$

18 $$\mathrm{R}=\frac{\sum_{\mathrm{i}-=1}^{\mathrm{N}}(\mathrm{x}-\widehat{\mathrm{x}})(\mathrm{y}-\widehat{\mathrm{x}})}{\sqrt{\sum_{\mathrm{i}=1}^{\mathrm{N}}{(\mathrm{x}-\widehat{\mathrm{x}})}^{2}\sum_{\mathrm{i}=1}^{\mathrm{N}}({\mathrm{y}-\widehat{\mathrm{y}})}^{2}}}$$

19 $$RMSE= \sqrt{\frac{\sum_{i=1}^{N}({x-y)}^{2}}{N}}$$

20 $$MAPE= \frac{1}{N}\left[\sum_{i=1}^{N}|\frac{x-y}{x}|\right]$$

where $x,y,\widehat{x}$ and $\widehat{y}$ are the actual, predicted, average actual and average predicted values, respectively.
